# Improving lung cancer screening: An equitable strategy through a tobacco treatment clinic

**DOI:** 10.1016/j.pmedr.2021.101558

**Published:** 2021-09-17

**Authors:** Panagis Galiatsatos, Raiza Schreiber, Kamala Green, Rohan Shah, Hans Lee, David Feller-Kopman, Lonny Yarmus, Jeffrey Thiboutot, Cheng Ting Lin, Norma Kanarek

**Affiliations:** a Division of Pulmonary and Critical Care Medicine, Department of Medicine, Johns Hopkins University School of Medicine, Baltimore, MD, United States; b The Johns Hopkins Tobacco Treatment Clinic, Johns Hopkins Bayview Medical Center, Baltimore, MD, United States; c Office of Diversity, Inclusion, and Health Equity, Johns Hopkins Medicine, Baltimore, MD, United States; d Sidney Kimmel Cancer Center, Department of Oncology, Johns Hopkins University School of Medicine; e Department of Radiology, Johns Hopkins Medicine, Baltimore, MD, United States; f The Ohio State University College of Medicine, Columbus, OH, United States; g Department of Environmental Health and Engineering, Johns Hopkins Bloomberg School of Public Health, Baltimore, MD, United States; h Baltimore City Health Department, Baltimore City, MD, United States

**Keywords:** Tobacco, Lung cancer screening, Health equity

## Abstract

Annual screening with low dose chest tomography has been adopted for those at high risk to aid in the early detection of lung cancer. In addition to screening, it is recommended that such persons receive evidence-based smoking-cessation. However, both lung cancer screening and evidence-based smoking-cessation strategies are underutilized in the US. We review the impact of a dedicated Tobacco Treatment Clinic (TTC), delivering evidence-based smoking cessation strategies, on lung cancer screening enrollment. Patients of the TTC, aged 50 years or older, having a minimum 20-pack-year smoking history were included. All patients had records reviewed to see if they had received lung cancer screening; if their lung cancer screening was achieved through the TTC, this was documented as “initial screening” versus “continued screening or surveillance”. Sociodemographic variables were collected as well. As for results, between January 2019 to February 2020, 92 patients enrolled in the TTC and fulfilled criteria for lung cancer screening. The mean age was 65.7 ± 8.3 years old, with 58 (63.0%) of the patients being female. Seventy-five (81.5%) patients were African American. Of the 92, 68 (73.9%) patients had lung cancer screening, with 51 patients receiving their first lung cancer screening scan through the TTC. In conclusion, through enrollment in a dedicated TTC, a significant proportion of patients were able to access lung cancer screening for the first time. Further, many of these patients were of minority status. Having a dedicated TTC may improve current health equity gaps in lung cancer screenings in certain US populations.

## 1 Introduction

In 2011, the National Lung Cancer Screening Trial reported that annual low-dose chest computer tomography (LDCT) led to a 20% reduction in lung cancer mortality for heavy smokers when compared to an annual chest x-ray (National Lung Screening Trial Research et al., 2011). Building off such evidence, the United States Preventative Services Task Force (USPSTF) issued its first recommendation for annual LDCT for adults 55–80 who have a 30 pack year smoking history and currently smoke or have quit within the past 15 years (Moyer and Force, 2014). Since the initial recommendation, updated guidelines by the USPSTF continue to emphasize lung cancer screenings of persons with a significant smoking history (Cheung et al., 2018, Richards et al., 2019). In accordance with the USPSTF’s recommendation for lung cancer screening, the American Cancer Society (ACS) recommends the use of LDCT for lung cancer screening in adults with a cigarette smoking history (Wender et al., 2013, Smith et al., 2018). With the aforementioned evidence and medical organizations’ emphasis on the clinical utility of screening, assurance of equitable implementation must be a priority to prevent lung cancer morbidity and mortality in persons with a smoking history.

However, despite such evidence, lung cancer screening has been underutilized in the US (Pham et al., 2020, Jemal and Fedewa, 2017, Soneji et al., 2017). For instance, only 4% of eligible patients undergo lung cancer screening (Sands et al., 2021), and 18% of current US adult smokers reported a discussion with their healthcare providers regarding lung cancer screening in the prior year (Soneji et al., 2020). Further, there are health disparities in regards to lung cancer screenings in the US, specifically amongst African Americans. African Americans are less likely to undergo screening, despite having a greater benefit in mortality reduction with lung cancer screening, as compared to whites (Carter-Harris et al., 2018, Japuntich et al., 2018, Aldrich et al., 2019). The difference in screening rates in African Americans may be multi-factorial, and include unawareness of the benefits of lung cancer screening to low socioeconomic status / lack of accesses (Japuntich et al., 2018, Aldrich et al., 2019); and as such, warrants a novel approach to improving lung cancer screening rates amongst this population.

Along with smoking cessation, lung cancer screening with LDCT is an effective preventative intervention in reducing lung cancer-related mortality (Jemal and Fedewa, 2017, Centers for Disease Control and Prevention. What Are the Risk Factors for Lung Cancer Atlanta, GA: Division of Cancer Prevention and Control, Centers for Disease Control and Prevention;, 2018, de Koning et al., 2020). Therefore, a clinical center that combines both efforts may improve lung cancer outcomes, especially if such a center is accessible by populations experiencing disproportionately higher rates of smoking. We aim to examine the utility of a tobacco treatment clinic that provides lung cancer screening, with specific interest in its ability to improve screening rates amongst minority populations.

## 2 Methods

### 2.1 Patient selection

All patients age 50 years old to 80 years old with a 20 pack-year smoking history17 (Wood et al., 2018); enrolled in the Tobacco Treatment Clinic at Johns Hopkins Bayview Medical Center were eligible for consideration for lung cancer screening. Patients would either receive a referral for an LDCT of their chest for either their first lung cancer screening or an appropriate follow-up or surveillance screen if a prior imaging raised concerns. If prior imaging was available, it was reviewed by the Tobacco Treatment Clinic staff prior to the patient’s appointment. The time period of patient selection was January 1, 2019, as this is when lung cancer screening services began at the Clinic. We opted to review up to February 29, 2020, as with the COVID-19 pandemic, much of our clinic transitioned into telemedicine visits, which would make the novelty of attempting same-day lung cancer screening challenging. Insight into the feasibility of lung cancer screenings during this public health crisis is warranted but will not be addressed with this review. The study was approved by Institutional Review Board at Johns Hopkins School of Medicine (IRB00282725) and all actions undertaken by the authors were in accordance with the Declaration of Helsinki.

All patients received an evidence-based model of chronic care for management of their tobacco dependence in an effort to achieve smoking cessation and, ultimately, tobacco dependence. Such management included counseling with or without pharmacological interventions, with weekly touch points via text-messaging or telephone calls (Leone et al., 2020, In: Smoking Cessation: A Report of the Surgeon General. Washington (DC), 2020). Return clinic visits occurred every 4–8 weeks during the first year of management; after the first year, clinical visits occur every 3-months until the patient achieves a consistency of tobacco independence. Counseling and touch points were coordinated by our physicians, tobacco treatment specialists, and nurse.

### 2.2 Lung cancer screening multi-disciplinary approach

The ability to conduct lung cancer screenings on all eligible patients in the Tobacco Treatment Clinic is performed through coordination with pulmonary medicine, radiology, and interventional pulmonary. As practice, we attempt to have all patients receive lung cancer screenings performed the same day as their clinical appointments. If insurance does not allow for LDCT to be conducted by our facilities, we provide the patient with a referral to obtain imaging at an insurance-approved facility.

**Fig. 1:**
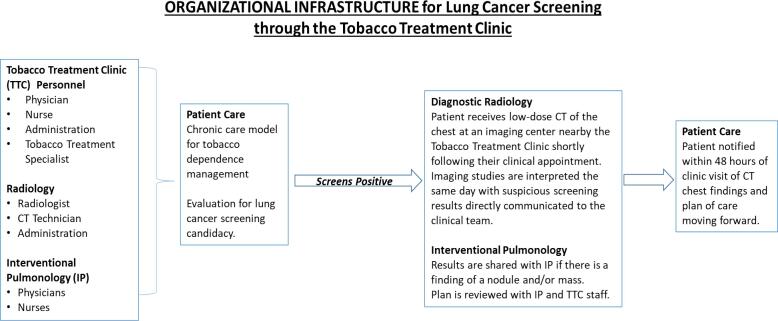
Multi-disciplinary workflow for lung cancer screenings.

Fig. 1 summarizes the process of the referral for lung cancer screening. Imaging findings were classified and reported using the Lung CT Screening Reporting and Data System developed by the American College of Radiology (ACoR, 2019). Interventional Pulmonary would assist in referring the patient to an oncology team depending on the finding of the biopsies. Note that parking accommodations are also provided for all patients receiving same-day lung cancer screening.

Each patient is reviewed the business-day prior to their clinical visit by the physicians and nurses of the tobacco treatment clinic. The review assesses age and imaging of their lungs. As mentioned before, each review explores if prior imaging is available of the patient’s lung, specifically, a computer topography scan of the chest. If the patient fulfills the screening age and lacks any imaging in our electronic medical records or has had recent imaging that is of concern that warrants follow-up, the patients would be flagged. Note that packs-per-year is documented during the actual clinic visit. A list of the flagged patients were emailed to the administrative personnel identified at the Department of Radiology, who in turn, would email by the end of the business day to confirm insurance eligibility for each patient. Meaning, which patients could obtain their lung cancer screening on-site at the hospital versus those that would have to be referred out.

During the clinic visit, if the flagged patient fulfills all criteria for lung cancer screening, at the end of the clinic session for tobacco dependence management, the provider discusses with the patient the benefit of lung cancer screening. For the patients who can undergo imaging on-site, a telephone call was placed to the imaging technicians to confirm availability. Due to the collaboration between the clinic and the Department of Radiology, there were saved slots for same-day lung cancer screenings; the telephone call to the technicians reaffirmed the availability. At that point, the patient completed their clinical session and went to perform their lung cancer screening. Total time between the clinical session and completion of lung cancer screening never exceeded 3-hours. For the patients who, due to insurance, could not receive same day on-site lung cancer screening, we discussed we would send an order for lung cancer screenings to the insurance-requested imaging site. Afterwards, with our clinic’s weekly phone calls to assess each patient’s journey in smoking cessation, we would review if the patient has completed their respective lung cancer screening. This follow-up was performed by the tobacco treatment clinic’s nurse.

Every patient that was flagged for lung cancer screening, and fulfilled the final variable (packs-per-year), underwent a conversation with the tobacco treatment clinic’s physician regarding lung cancer screening. Specifically, the physician discussed the current evidence of the benefit of lung cancer screening, as well as the harms of screening, and how it is performed. Further, the physician described the process if the lung cancer screening identified anything concerning: from continuing imaging to invasive procedures, such as a bronchoscopy with biopsy to the potential for need for thoracic surgery. The patient and physician underwent a shared decision making process, where the patient would share their thoughts and concerns, ultimately deciding on whether to undergo screening and if they desired to undergo the screening same day (if eligible) or wait for another time to perform the screening.

Note that for patients who had a finding on their lung cancer screening, interventional pulmonary (Fig. 1) would weigh in as to the need to have the patients seen in their Pulmonary Nodule Clinic or sent for invasive investigations (such a bronchoscopy and/or surgery). Such decisions were made within 24-hours of lung cancer screening completion. If the lung-cancer screening identified no significant findings, the patients’ lung cancer screening services would continue to be performed by the Tobacco Treatment Clinic, as recommended by current evidence (U.S. Department of Health and Human Services, 2021).

### 2.3 Outcome measurements and statistical analysis

The primary outcome was assessing if a patient received a lung cancer screening referral, for either initial screening or appropriate follow-up, through the Tobacco Treatment Clinic. Sociodemographic variables collected included age, gender, race, and area deprivation index (a contextual-level factor that is composed of several variables from the US Census and American Community Survey) (Kind and Buckingham, 2018). Co-morbidities were also collected for each patient. As for tobacco history, variables collected included age of initiation of smoking, packs-per-year, and if a patient has never attempted a cessation effort. Variables are presented as mean ± standard deviation or median (interquartile range, IQR) where appropriate.

## 3 Results

Between January 2019 to February 2020, 92 patients attended the Tobacco Treatment Clinic for in-person clinical visits and qualified for lung cancer screening. These patients were referred by various medical services: 51 internal medicine referrals, 24 surgical referrals, 6 pediatric referrals, and 11 self-referrals. From the internal medicine referrals, 19 were from pulmonary, 14 were from cardiology, 11 from general internal medicine, and 7 from infectious disease. Of the surgical referrals, 9 were from bariatric surgery, 7 were from plastic surgery, and 5 were from orthopedics. All 6 pediatric referrals were for patient’s parents or guardians.

The mean age of the 92 patients was 65.7 ± 8.3 years old, with 58 (63.0%) of the patients being female, and the majority, 75 (81.5%) patients, were African Americans. From a contextual-level status, the median ADI of the 92 patients is 68 (IQR 42, 94), ranging from 32 to 99 (1 = worst, 100 = best). The most common morbidities of the 92 patients included hypertension (58 patients), chronic obstructive pulmonary disease (COPD) (36 patients), and diabetes (32 patients). The mean lifetime pack-years was 42.3 ± 13.4, ranging from 34 pack-years to 80 pack-years. A summary of sociodemographic variables and pre-existing conditions is found in Table 1.

**Table 1:** Demographic data of the 92 patients enrolled in the Tobacco Treatment Clinic.

**Variable**	
**Age (years)**	65.7 ± 8.3
**Female (%)**	58 (63.0)
**African American (%)**	75 (81.5)
**Area Deprivation Index (IQR)**	68 (42, 94)
**Pre-existing Conditions (%)**	
COPD	36 (39.1)
Hypertension	58 (63.0)
Congestive Heart Failure	10 (10.9)
Diabetes	32 (34.8)
HIV	6 (6.5)
Cirrhosis	4 (4.3)
Chronic Kidney Disease	11 (12.0)
None	10 (9.2)
**Packs-per-Years**	42.3 ± 13.4
**Age of Smoking Initiation (years)**	14.3 ± 3.6
**Never Attempted Smoking Cessation (%)**	16 (17.4)

COPD = Chronic Obstructive Lung Disease; HIV = Human Immunodeficiency Virus.

### 3.1 Lung cancer screening & surveillance results

Of the 92 patients in the Tobacco Treatment Clinic, 24 patients had already received lung cancer screening. The remaining 68 (73.9%) patients received appropriate lung cancer screening as a direct result of the Clinic. Of these 68 patients, 51 (75.0%) received their first lung cancer screening, while 17 (25.0%) received appropriate subsequent or surveillance screening. The mean age of these patients was 57.3 ± 4.6 years old, and 40 (58.8%) of the 68 patients were females. Further, 54 (79.4%) of the 68 patients were African American. A summary of these patients is found in Table 2. Of the 24 patients who had already achieved appropriate lung cancer screening prior to enrollment in the TTC, 14 were patients referred by pulmonary, 7 by cardiology, and 3 by general internal medicine.

**Table 2:** Demographic data and imaging outcomes of the 68 patients who received lung cancer screening through the Tobacco Treatment Clinic.

**Variable**	
**Age (years)**	57.3 ± 4.6
**Female (%)**	40 (58.8)
**African American (%)**	54 (79.4)
**Area Deprivation Index (IQR)**	72 (58, 96)
**First Lung Cancer Screening (%)**	51 (75.0)
**Follow-up or Surveillance Screening (%)**	17 (25)
Concerning findings from prior imaging	8 (11.8)
Time since previous imaging (yeas)	3.2 ± 0.8
**Same-day Lung Cancer Screening (%)**	49 (72.1)
**Referral for Biopsy (%)**	3 (4.4)

Seventeen patients received follow-up or surveillance imaging as a direct result of concerning findings from a prior chest CT scan. Of these seventeen patients, 4 patients had received imaging from a prior clinician for lung cancer screening purposes. The remaining 13 patients received imaging for prior active symptoms necessitating lung imaging (symptoms such as dyspnea, frequent cough, chest pain). The mean time since previous imaging was 3.2 ± 0.8 years, with a range of 6-months to 5.4 years. Eight patients had concerning findings from prior imaging, specifically pulmonary nodules (range 4 mm to 1.2 cm), ground glass opacities, or mediastinal lymphadenopathy. Three patients were referred to Interventional Pulmonary for consideration of biopsy.

In regard to the feasibility of the referrals for and completion of lung cancer screening, 49 (72.1%) of the 68 patients were able to complete imaging on the same day as the TTC visit. The remaining 19 patients completed their imaging at non-affiliated radiographic offices, as per insurance mandates. Time to completion of lung cancer screening for the 19 patients ranged from 17 days to 64 days. Therefore, all eligible patients, as identified by the TTC, completed lung cancer screening.

## 4 Discussion

Between 2019 and early 2020, seventy-four percent of patients who qualified for lung cancer screening received such screening through our Tobacco Treatment Clinic, significantly higher than the U.S. average of 4% (Sands et al., 2021). The majority of such patients in our study were African American and/or from socioeconomically disadvantaged neighborhoods, both of which are sociodemographic variables known to result in lower lung cancer screenings (Jemal and Fedewa, 2017). Further, the majority of patients who qualified for lung cancer screening had such screening performed the same-day they received smoking cessation counseling and management. Therefore, given the findings from our tobacco treatment clinic on lung cancer screenings, such an approach may be advantageous to impact differences in lung cancer mortality amongst populations disproportionately impacted by lung cancer.

Improving lung cancer screening amongst patient populations who suffer disproportionate lung cancer outcomes is a priority. For instance, Lake et al found that at an urban academic medical center’s lung cancer screening program, African Americans still had lower odds of receiving lung cancer screenings as compared to whites (Lake et al., 2020). Further, the authors found that neighborhood socioeconomics impacted lung cancer screenings, as they found it was marginally correlated with screening completion (Lake et al., 2020). Of note, race and neighborhood composition have also been identified as factors associated with lower rates of smoking cessation (Lee and Kahende, 2007, Trinidad et al., 2011). In fact, many of the variables associated with low completion of lung cancer screening (Wang et al., 2019) are similar to poor smoking cessation rates (Trinidad et al., 2011) in certain minority populations. Therefore, addressing both issues simultaneously, as achieved in our Tobacco Treatment Clinic, holds promise as an efficient health equity strategy.

Currently, 14.0% of the US adult population identifies as current smokers, with higher rates seen across certain sociodemographics, such as low income and minority race (The Centers for Disease Control and Prevention, 2021). However, in Baltimore City, neighborhoods of low socioeconomic status and high minority populations exceed the national average of US adult smokers, with over 50 census blocks in Baltimore City having active smoking rates between 30.7% and 43.7% (Baltimore City Health Department, 2016). And these same regions in Baltimore City have the highest lung cancer mortality rates, as compared to more affluent neighborhoods (Baltimore City Health Department, 2016). Our patient cohort reflects such sociodemographics, with our cohort high in minorities and a majority residing in socioeconomically disadvantaged neighborhoods. Therefore, assuring an effective and equitable strategy to reduce lung cancer morbidity and mortality may be achieved by providing services that first aim to mitigate the risk factor contributing significantly to such disproportionate lung cancer development: smoking.

Evidence to support reduction of lung cancer mortality by early lung cancer detection through screening via LDCT supports simultaneous implementation of smoking cessation efforts (Cheung et al., 2018, , xxxx, Blackmon and Feinglass, 2015, Printz, 2020). Smoking cessation in addition to lung cancer screening extends the endpoints of early cancer diagnosis and cancer mortality mitigation, and by extension towards risk reduction in many other diseases (Steliga and Yang, 2019). Further, having the focus of a clinical encounter be specifically for smoking cessation, as achieved by a tobacco treatment clinic, may help to mitigate any impact of stigma and shame that often delays a patient who smokes from receiving evidence based care for treatment of tobacco dependence (Curry et al., 2013, Carter-Harris et al., 2014). Such clinical approaches, with a primary focus on smoking cessation supplemented with assurance of lung cancer screening achievement, likely resulted in the high completion of lung cancer screening in our cohort of patients identified as appropriate candidates. The success of the lung cancer screening completion extended to all patients sent for referral, even in those in whom same day completion of the screening could not be achieved. Future lung cancer screening programs should consider emphasizing smoking cessation, not as an adjunct, but potentially as the main discussion in an effort to allow the individual to comfortably approach lung cancer screening.

This study should be viewed in the context of its limitations. First, it is unclear if screening and completion of low-dose computed tomography for lung cancer has any immediate influence on the management of tobacco dependence. As smoking cessation may take time, with the journey to tobacco independence often warranting an extension of treatment for months to years (Galiatsatos et al., 2021), assessing the impact of lung cancer screening to the overall goal of tobacco independence warrants future evaluation. Second, it is unclear if a tobacco treatment clinic serving an urban population and providing same-day lung cancer screening can be generalized to other communities. Efforts to mitigate lung cancer’s impact in rural communities with high rates of smoking should be a primary focus of future equity studies, with consideration for tobacco treatment clinics as a potential clinical tool. Finally, the success of lung cancer screening achievement was one fulfilled due to a multi-disciplinary approach, from pulmonary medicine to nursing to interventionists to radiology. This as well may be difficult to generalize in other medical centers; however, we believe the value of the outcome as seen in this small cohort warrants a commitment for such similar approaches in other medical centers to consider replicating.

A dedicated tobacco treatment clinic offering lung cancer screening services in a supportive manner may prove advantageous towards improving lung cancer screening in patients committed to intensive tobacco treatment. Through such a clinic, reaching marginalized patient populations may result in mitigating health disparities in both smoking cessation and lung cancer mortality. Given our study is limited by a small cohort of patients, future investigations should evaluate the clinical utility of such a multi-disciplinary effort on both smoking cessation rates and reduction in lung cancer mortality. Overall, a dedicated tobacco treatment clinic may be appropriate to improve the current health equity gaps in lung cancer screenings in certain US populations.

## Financial Disclosure

Dr Kanarek is funded in part by NCI Regional Oncology Research Center (5P30 CA006973-57). Dr. Galiatsatos and Kanarek are funded in part by the MD Cigarette Restitution Fund Research Grant at Johns Hopkins.

## Declaration of Competing Interest

The authors declare that they have no known competing financial interests or personal relationships that could have appeared to influence the work reported in this paper.
